# Supplementary figures and images for: Immediate Patient Satisfaction with Dental Esthetics After Endodontic and Prosthodontic Treatment of Dental Dyschromia
Source: Dent J (Basel). 2025 Jan 20;13(1):44. doi: 10.3390/dj13010044 (PMC11764403; doi:10.3390/dj13010044)

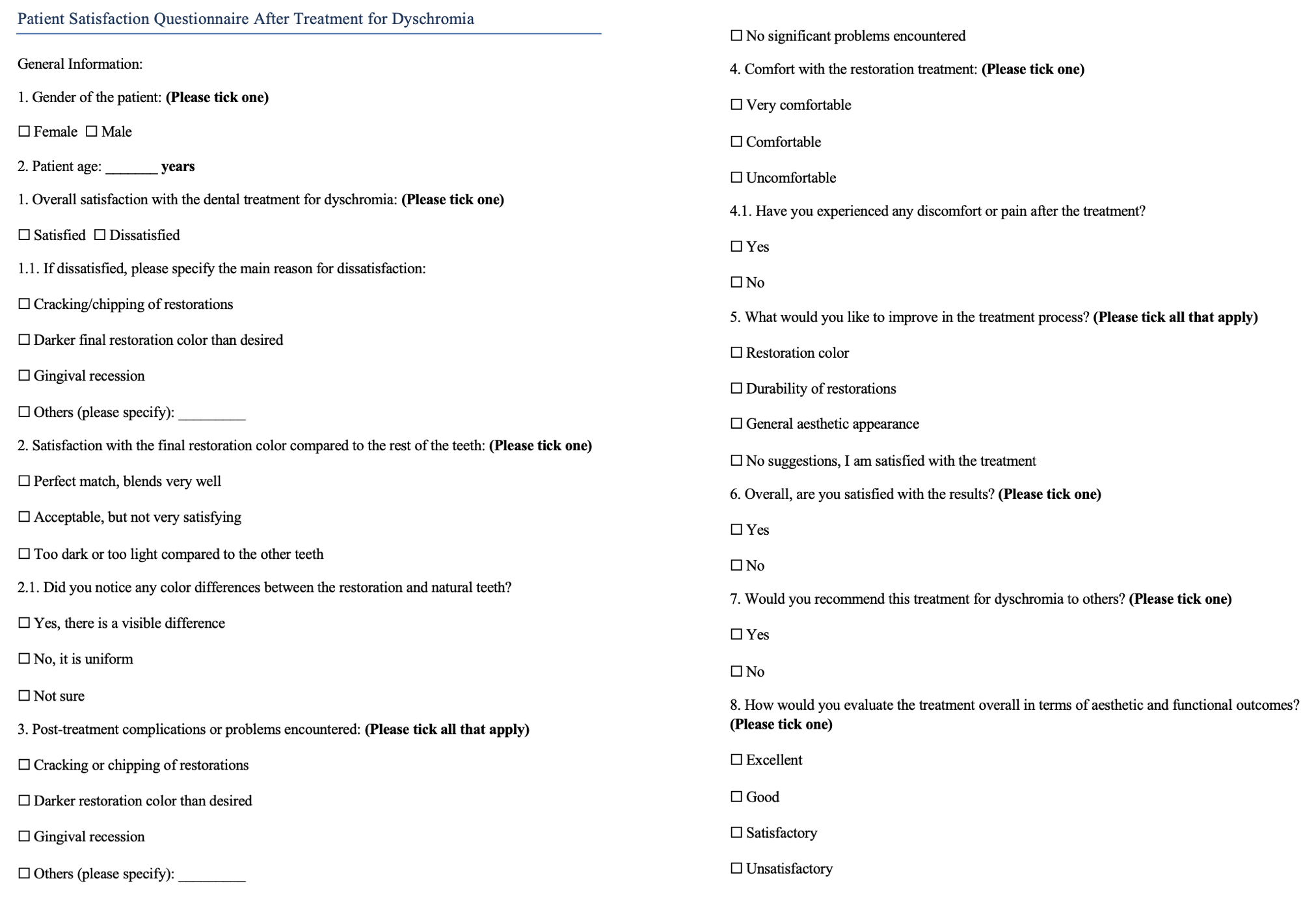

Supplement: Supplementary file 1 [file dentistry-13-00044-s001.zip › dentistry-3344310-supplementary.png]
